# Nursing workload associated with the frequency of multidisciplinary rounds: a cross-sectional study

**DOI:** 10.5935/0103-507X.20210008

**Published:** 2021

**Authors:** Maria Luiza Borges, Pedro Caruso, Antonio Paulo Nassar Júnior

**Affiliations:** 1 Intensive Care Unit, A.C. Camargo Cancer Center - São Paulo (SP), Brazil.; 2 Pulmonary Division, Heart Institute, Hospital das Clínicas, Faculdade de Medicina, Universidade de São Paulo - São Paulo (SP), Brazil.

**Keywords:** Critical care, Multidisciplinary communication, Staff engagement, Workload, Patient care team, Cuidados críticos, Comunicação interdisciplinar, Engajamento no trabalho, Carga de trabalho, Equipe de assistência ao paciente

## Abstract

**Objective:**

To assess the frequency of multidisciplinary rounds during ICU days, to evaluate the participation of diverse healthcare professionals, to identify the reasons why rounds were not performed on specific days, and whether bed occupancy rate and nurse workload were associated with the conduction of multidisciplinary rounds.

**Methods:**

We performed a cross-sectional study to assess the frequency of multidisciplinary rounds in four intensive care units in a cancer center. We also collected data on rates of professional participation, reasons for not performing rounds when they did not occur, and daily bed occupancy rates and assessed nurse workload by measuring the Nursing Activity Score.

**Results:**

Rounds were conducted on 595 (65.8%) of 889 surveyed intensive care unit days. Nurses, physicians, respiratory therapists, pharmacists, and infection control practitioners participated most often. Rounds did not occur due to admission of new patients at the scheduled time (136; 44.7%) and involvement of nurses in activities unrelated to patients’ care (97; 31.9%). In multivariate analysis, higher Nursing Activity Scores were associated with greater odds of conducting multidisciplinary rounds (OR = 1.06; 95%CI 1.04 - 1.10; p < 0.01), whereas bed occupancy rates were not (OR = 0.99; 95%CI 0.97 - 1.00; p = 0.18).

**Conclusion:**

Multidisciplinary rounds were conducted on less than two-thirds of surveyed intensive care unit days. Many rounds were cancelled due to activities unrelated to patient care. Unexpectedly, increased workload was associated with higher odds of conducting rounds. Workload is a possible trigger to discuss daily goals to improve patient outcomes and to enhance the effectiveness of multidisciplinary teams.

## Abstract (pt)

**Objetivo:**

Avaliar a frequência de visitas multidisciplinares durante a estadia na unidade de terapia intensiva e a participação dos diferentes profissionais da unidade, identificar as razões pelas quais as visitas não foram realizadas em dias específicos e se a taxa de ocupação e a carga de trabalho da enfermagem estavam associadas com a realização de visitas multidisciplinares.

**Métodos:**

Realizamos um estudo transversal para avaliar a frequência de visitas multidisciplinares em quatro unidades de terapia intensiva localizadas em um centro para tratamento de câncer. Colhemos também dados referentes à participação de profissionais, a razões para não realização das visitas nos casos em que elas não ocorriam e a taxas diárias de ocupação de leitos, assim como avaliamos a carga de trabalho da enfermagem por meio do Nursing Activity Score.

**Resultados:**

Foram conduzidas visitas multidisciplinares em 595 (65,8%) dos 889 dias de unidade de terapia intensiva avaliados. Mais frequentemente tomaram parte dessas visitas enfermeiros, médicos, fisioterapeutas respiratórios e profissionais ligados ao controle de infecções. As visitas não ocorreram em razão da admissão de novos pacientes no horário programado para a visita (136; 44,7%) e do envolvimento dos enfermeiros em atividades não relacionadas ao cuidado de pacientes (97; 31,9%). Na análise multivariada, níveis mais elevados do Nursing Activity Score se associaram com maior tendência à realização de visitas multidisciplinares (RC = 1,06; IC95% 1,04 - 1,10; p < 0,01), enquanto as taxas de ocupação não tiveram essa associação (RC = 0,99; IC95% 0,97 - 1,00; p = 0,18).

**Conclusão:**

Realizaram-se visitas multidisciplinares em menos de dois terços dos dias de unidade de terapia intensiva pesquisados. Muitas das visitas foram canceladas em razão de atividades não relacionadas aos cuidados com o paciente. A carga de trabalho é um possível gatilho para discussão dos alvos do dia para melhorar os desfechos dos pacientes e incrementar a efetividade das equipes multidisciplinares.

## INTRODUCTION

Multidisciplinary teams are essential for the care of critically ill patients. Daily multidisciplinary rounds are correlated with numerous positive outcomes, such as the implementation of sedation protocols,^(1)^ earlier mobilization,^(2)^ fewer adverse drug events,^(3)^ reduced use of invasive devices^(4)^ and lower mortality.^(5)^ In addition, multidisciplinary rounds using daily goal checklists are associated with improved perceptions of work and patient safety climates.^(6)^ Multidisciplinary rounds may be even more important in strained settings.^(7)^

However, 20 - 30% of intensive care units (ICUs) surveyed in numerous studies do not perform multidisciplinary rounds.^(8,9)^ Given that professionals from diverse disciplinary backgrounds bring alternative perspectives that can lead to vastly different conclusions regarding specific aspects of patient care,^(10,11)^ multidisciplinary collaboration should be encouraged. In addition, communication failures may cause adverse events and prolong ICU lengths of stay.^(12)^ Thus, the implementation of daily multidisciplinary rounds should be a top priority of quality improvement programs in ICUs.

We aimed to assess the frequency of multidisciplinary rounds during ICU days, to evaluate the participation of diverse professional disciplines responsible for critical care, to identify the reasons why rounds were not performed on specific days, and to identify whether two measures of ICU capacity strain,^(13)^ e.g., bed occupancy rate and nurse workload, were associated with the conduction of multidisciplinary rounds.

## METHODS

We conducted a cross-sectional study in four ICUs in an academic cancer center from October 2017 to August 2018. The study was approved by the Ethics Committee (number 2,430/17). Due to the observational study design, the requirement for informed consent was waived. We followed the Strengthening the Reporting of Observational Studies in Epidemiology guidelines.^(14)^

Our study was conducted in one of two unconnected main hospital buildings that has four ten-bed mixed medical-surgical ICUs. Patients can be admitted to any of the ICUs. During the morning shift, there was one physician and one nurse every five beds, a respiratory therapist every ten beds, and a nurse technician every two beds. There is also a pharmacist for every ten to 20 beds. Depending on the on-call schedule, the pharmacist may also be responsible for ward beds.

Multidisciplinary rounds are scheduled daily from 11 to 12 a.m. from Monday through Friday. The participation of physicians, nurses and respiratory therapists is mandatory. Pharmacists, nutritionists, psychologists, and infection control practitioners are invited, but their participation is optional. Rounds are conducted at nurses’ stations rather than at the patients’ bedsides. In general, the physician in charge presents each patient’s clinical status and proposes a diagnostic and therapeutic plan. All professionals discussed critical medical problems from their perspectives and suggested management strategies. Interventions must be documented and checked in electronic health records. Briefly, these activities can be summarized as requests for imaging and laboratory tests; drug reconciliation and dosing changes; withdrawal of invasive devices, such as central venous and urinary catheters; patient mobilization; and specialty consultations.

Although multidisciplinary rounds are considered part of the morning shift, they may be cancelled at the request of nurses or physicians due to emergent tasks that must be completed during the same time frame, such as patient admissions, transfers, or the performance of invasive procedures.

A nurse supervisor assessed daily whether multidisciplinary rounds were conducted in each of the four ICUs. If rounds were performed, she documented which professionals participated. Additionally, for all study days, she assessed the bed occupancy rate of each ICU and the Nursing Activity Score (NAS)^(15)^ of all patients to measure nurse workload. A daily mean NAS for each ICU was calculated by adding the NAS of each patient and dividing by the number of patients hospitalized in the particular ICU on that day. If rounds were not performed, she asked nurses in charge why rounds did not occur and recorded the reason. The reasons were categorized as follows: admission of a new patient during the ICU round schedule time, bedside procedures being performed by the physician on charge at round schedule time, nurses involved in activities unrelated to patients’ care (administrative or educational activities), and other.

We retrieved data of patients admitted during the study period from electronic medical records. We collected the following data: age, sex, type of cancer (solid locoregional, solid metastatic, hematologic or no cancer/remission > 5 years), Eastern Cooperative Oncology Group Performance Status (ECOG PS), type of admission (medical, elective or urgent surgery), reason for admission, Simplified Acute Physiology Score (SAPS) 3, ICU outcomes (alive, dead or transferred to another hospital) and length of ICU stay.

The study’s outcome was the measurement of the frequency of conducting multidisciplinary rounds during ICU days over the study period.

### Statistical analysis

Categorical variables are presented as absolute numbers and percentages. Continuous variables are presented as the means and standard deviations.

We tested whether bed occupancy and mean NAS were associated with conduction of multidisciplinary rounds by performing Student’s t-test. We also performed a logistic regression with bed occupancy and mean NAS as independent variables and accomplishment of a multidisciplinary round as the dependent variable. Odds ratios (ORs) and 95% confidence intervals (95%CIs) were calculated for both variables included in the model. A p value of < 0.05 was considered significant. All analyses were performed using Statistical Package for Social Sciences (SPSS), version 21 (IBM Corporation, Armonk, NY, United States).

## RESULTS

**Figure 1:**
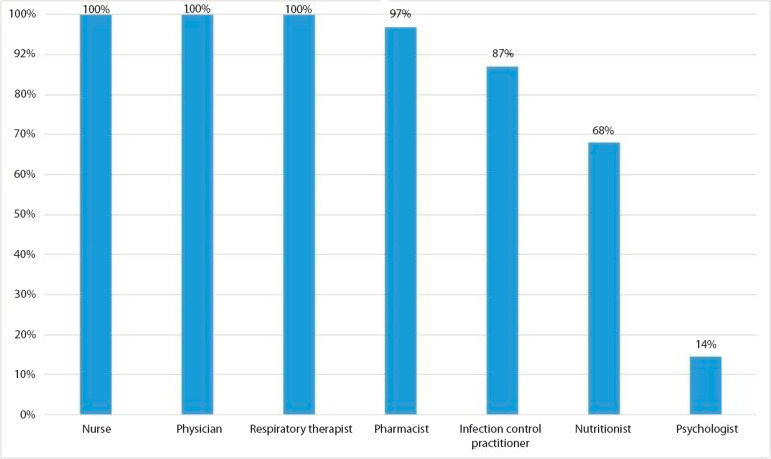
Professional participation in multidisciplinary rounds.

There were 223 days with scheduled multidisciplinary rounds during the study period. However, one unit was closed for 3 days for equipment maintenance. Consequently, we assessed the frequency of multidisciplinary rounds on 889 ICU days. Multidisciplinary rounds were conducted in 585 (65.8%) of these opportunities. Nurses, physicians and respiratory therapists participated in all rounds. Pharmacists and infection control practitioners were other frequent participants (Figure 1).

**Figure 2:**
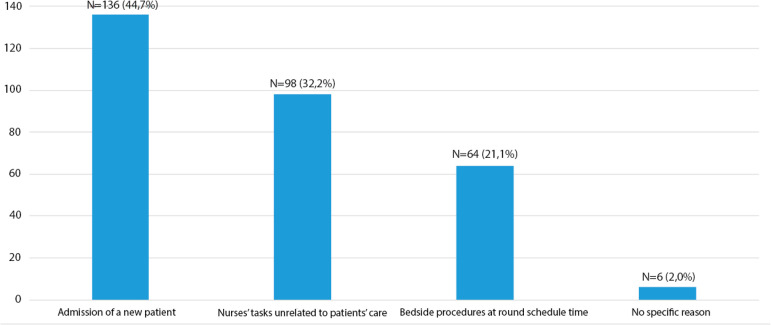
Reasons for multidisciplinary round cancellation.

The main reason for not performing multidisciplinary rounds was an admission of patients in the round scheduled time (136, 44.7%). On six occasions (2.6%), multidisciplinary rounds did not occur due to “no specific reason” according to the nurses in charge. The involvement of nurses in activities unrelated to patient care led to the cancellation of 97 (31.9%) rounds (Figure 2).

A total of 3,096 patients were admitted during the study period. The patients were predominantly male (1629, 52.6%), had a mean age of 61 (± 15.1) years and had predominantly solid cancers (2,790; 90.1%). Medical reasons (1,608, 51.9%) were more common than surgical admissions. Data on previous performance status were available for 2,298 (74.2%) patients, of whom 1,494 (65.0%) had absent or minor impairment status. A total of 275 (8.9%) patients died at ICU discharge (Table 1).

**Table 1:** Characteristics of patients admitted during the study period

Variable	
Age	61.0±15.1
Female sex	1.467 (47.4)
Type of admission	
Medical	1.608 (51.9)
Elective surgery	1.310 (42.3)
Urgent surgery	176 (5.7)
Source of admission	
Surgical room	1.476 (47.7)
Emergency department	818 (26.4)
Wards	775 (25.0)
Another hospital	25 (0.8)
Reason for admission	
Postoperative monitoring	1.450 (46.8)
Sepsis/septic shock	466 (15.1)
Neurological disorders	163 (5.3)
Cardiovascular disorders	148 (4.8)
Renal and metabolic disorders	78 (2.6)
Type of tumor	
Solid loco regional	1.384 (44.7)
Solid metastatic	1.406 (45.4)
Hematologic	206 (6.7)
No cancer or remission > 5 years	98 (3.2)
SAPS 3 points	54.9±16.6
Performance status*	
No or minor impairment (ECOG 0 or 1)	1.494 (65.0)
Moderate impairment (ECOG 2)	442 (19.2)
Severe impairment or bedridden (ECOG 3 or 4)	362 (15.8)
ICU discharge outcomes	275 (8.9)
Alive	2.818 (90.0)
Dead	275 (8.9)
Transferred to another hospital	3 (0.1)
Length of ICU stay, days	3.0±4.0

SAPS - Simplified Acute Physiology Score; ECOG - Eastern Cooperative Oncology Group; ICU - intensive care unit.

* Data on performance status were not available for 798 (25.8%) patients. Results expressed as mean ± SD or n (%).

The mean patient NAS was higher on days with rounds compared with those without rounds (86.2 ± 5 *versus* 84.8 ± 4.3; p < 0.01). On the other hand, bed occupancy rates did not differ on days with or without rounds (93 ± 9.7 *versus* 93.5 ± 9.8%, p = 0.45). On logistic regression, mean patient NAS was independently associated with the conduction of multidisciplinary rounds (OR = 1.07; 95%CI 1.04 - 1.10; p < 0.01), whereas bed occupancy rates were not (OR = 0.99; 95%CI 0.97 - 1.00; p = 0.18).

## DISCUSSION

Our study showed that multidisciplinary rounds in ICUs were conducted in less than two-thirds of ICU days. As expected, nurses, physicians, and respiratory therapists participated in all rounds, and pharmacists and infection control practitioners also participated often. Rounds were more frequent on higher nurse workload days. New patients’ admission and nurses’ involvement in activities unrelated to patients’ care were the main reasons for not performing multidisciplinary rounds.

Multidisciplinary rounds are essential to the care of critically ill patients because professionals from diverse disciplines have varied perceptions and recognize different aspects of medical problems.^(10,11)^ Multidisciplinary rounds are associated with positive outcomes for both patients^(1,2,5)^ and interprofessional teams because they increase collaboration and understanding of daily goals,^(12)^ facilitate the sharing of both similar and complementary insights,^(16)^ enable consensus-based decision making, and reduce conflicts within teams.^(7)^ The fact that multidisciplinary rounds were not conducted in greater than one-third of scheduled days is cause for concern. A strategy for the reduction of competing tasks during rounds should be addressed as a top priority.^(17)^

A positive finding of this study was the frequent participation of pharmacists on rounds. Pharmacist participation is associated with reduced mortality rates, lengths of ICU stay, and adverse drug events.^(3)^

Our finding of greater odds of conducting multidisciplinary rounds on days of higher nurse workload was unexpected. Because rounds may be disrupted by emergent multitasks during scheduled rounding time, we believed that rounds would be less likely during higher nurse workload days. The opposite finding may be due to a team perception that the care of patients with more critical illnesses generates higher workloads and, consequently, demands multidisciplinary discussions to establish daily goals. Nurse workload is one of the indicators of ICU capacity strain^(13)^ and is associated with higher rates of burnout.^(18,19)^ In addition, high workload to nurse ratios are associated with increased mortality.^(20)^ We suggest that nurse workload should be a trigger to discuss daily goals to improve patient outcomes, reduce risk, and enhance the effectiveness of multidisciplinary teams.

This study has both strengths and limitations. This was a pragmatic study that aimed to assess the frequency of multidisciplinary rounds and its association with bed occupancy rates and nurse workload. To reach these aims, our study covered almost 9,000 patient/days in 889 possible encounters. On the other hand, it was a single center study, and our staffing model may not reflect those used in many centers. Additionally, we did not address differences in patient outcomes, achievement of daily goals, or measures of staff wellbeing related to the conduction of multidisciplinary rounds.

## CONCLUSION

Multidisciplinary rounds were conducted in only 65.8% of scheduled intensive care unit days. However, the professionals most responsible for directing patient care participated regularly. Rounds occurred more frequently on days of higher nurse workload. Admission of new patients and nurses’ tasks unrelated to patients’ care were the main reasons for not performing a multidisciplinary round. Future studies should focus on strategies to identify and decrease tasks that decrease conduction and/or participation in multidisciplinary rounds and to assess whether daily goals are achieved.
